# A report on SARS-CoV-2 first wave in Ecuador: drug consumption dynamics

**DOI:** 10.3389/fphar.2023.1197973

**Published:** 2023-06-14

**Authors:** Andrea Orellana-Manzano, Fernanda B. Cordeiro, Andrea Garcia-Angulo, Elizabeth Centeno, María José Vizcaíno-Tumbaco, Sebastián Poveda, Ricardo Murillo, Derly Andrade-Molina, Mariuxi Mirabá, Saurabh Mehta, Washington Cárdenas

**Affiliations:** ^1^ Laboratorio Para Investigaciones Biomédicas, Facultad de Ciencias de la Vida, Escuela Superior Politécnica del Litoral (ESPOL), Guayaquil, Ecuador; ^2^ Facultad de Ciencias Naturales y Matemáticas, Escuela Superior Politécnica del Litoral (ESPOL), Guayaquil, Ecuador; ^3^ Division of Nutritional Sciences, Cornell University, Ithaca, NY, United States; ^4^ Laboratorio de Ciencias Omicas, Facultad de Ciencias de la Salud, Universidad Espíritu Santo, Samborondon, Ecuador

**Keywords:** SARS-CoV-2, COVID-19, Ecuador, drug consumption, self-medication, public health

## Abstract

**Introduction:** The first COVID-19 wave in Ecuador started in March 2020 and extended until November. Several types of drugs have been proposed as a potential treatment during this period, and some affected people have self-medicated.

**Method:** A retrospective study was conducted with 10,175 individuals who underwent RT-PCR tests for SARS-CoV-2 from July to November 2020. We compared the number of positive and negative cases in Ecuador with symptoms and drug consumption. The Chi-square test of independence compared clinical and demographic data and PCR test results. Odds ratios analyzed drug consumption dynamics.

**Results:** Of 10,175 cases, 570 were positive for COVID-19, while 9,605 were negative. In positive cases, there was no association between the RT-PCR result and sex, age, or comorbidities. When considering demographic data, Cotopaxi and Napo had the highest rates of positive cases (25.7% and 18.8%, respectively). Manabí, Santa Elena, and Guayas regions had fewer than 10% positive cases. The Drug consumption dynamic analysis showed that negative COVID-19 cases presented higher drug consumption than positive cases. In both groups, the most consumed medication was acetaminophen. Acetaminophen and Antihistamines had higher odds of consumption in positive PCR cases than in negative. Symptoms like fever and cough were more related to positive RT-PCR results.

**Conclusion:** The first COVID-19 wave in Ecuador has affected the provinces differently. At a national level, the consumption of drugs has been highly associated with self-medication.

## Introduction

The widely used practice of self-medication (SM) has both advantages to patients and the healthcare system (Hughes et al., 2001), yet unmonitored and ill-informed may pose a significant risk to one’s health. With the advance of COVID-19 worldwide, the interest and practice in SM increased in the general population among healthcare workers (Molento, 2020; Onchonga et al., 2020; Orellana Manzano et al., 2021). A recent review of evidence from eight countries on the practice of SM for the prevention and treatment of COVID-19 showed a prevalence ranging from 4% to 88.3% in the general population and, among healthcare workers, 33.9%–51.3% (Quincho-Lopez et al., 2021). However, the prevalence of SM and drug consumption practices for COVID-19 remains to be discovered in many countries, including Ecuador.

The observed increased trend in the practice of SM may be associated with the healthcare infrastructure, which was severely affected by insufficient supplies, personnel, and equipment during the COVID-19 response (Onchonga et al., 2020). Many people avoided seeking medical assistance because of how overwhelmed the health centres wereand their concerns about getting sick (Bennadi, 2013). As a result, SM practices were widely used as a prevention method and to alleviate any symptoms or discomfort. The most common reasons for self-medicating during the pandemic included treatment of fever, emergency illness, distance from a healthcare facility, fear of stigmatization, affordability of medicines, and the belief that medical attention was unnecessary (Hughes et al., 2001; Quincho-Lopez et al., 2021).

Since COVID-19 was declared a pandemic, various organizations have collaborated to search for treatments for SARS-CoV-2 infection (Agencia Nacional de Regulación, 2020). The World Health Organization (World Health Organization, 2020) trials showed sporadic remdesivir, hydroxychloroquine, lopinavir, and interferon success (Dyer, 2020). Although the scientific information available was clear about the inefficiency of such treatments for COVID-19, many health professionals and patients have continued to use these drugs to treat and prevent COVID-19, increasing the risks and complications of potential drug abuse (Torijano Casalengua et al., 2021).

Among the most widely used drugs worldwide for COVID-19 are analgesics, anti-inflammatories, antiretrovirals, antibiotics, and ivermectin (Quispe-Cañari et al., 2021). In Ecuador, indigenous communities from the Amazon region have used acetaminophen and ibuprofen for symptom relief. They prefer natural plant-based medicine despite insufficient evidence that medicinal plants are effective against COVID-19 (Montaño, 2020).

This study aimed to determine the prevalence of COVID-19 infection and self-reported drug consumption practices during the first SARS-CoV-2 wave in Ecuador.

## Methods

This study was conducted during the first wave of SARS-CoV-2 in Ecuador, from July to November 2020. The study has been approved by the “Comité Nacional Expedito para Investigación sobre COVID-19” under protocol No. 024-2020. All participants signed informed consent before the interview and the PCR test for COVID-19.

### Study design

**FIGURE 1:**
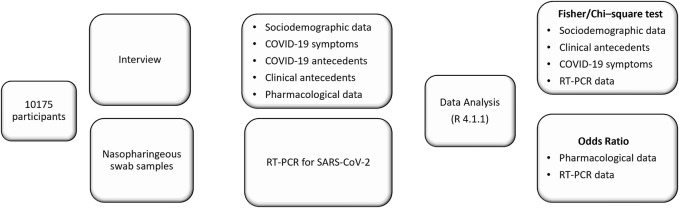
Graphic workflow summarizing different phases of the study.

Trained healthcare professionals interviewed participants to obtain general information based on demographic and clinical data before the COVID-19 RT-PCR test. The interview was divided into five sections: 1. Demographic data, including age, sex, address, city, and the province where they lived during the first wave, 2. COVID-19 symptoms and dates when symptoms first began, 3. History of prior COVID-19 positive test results and whether they consulted medical attention, 4. Clinical and past medical history, 5. Drug consumption for COVID-19 symptoms during the 14 days before the RT-PCR test (Figure 1).

The Biomedical Research Laboratory provided assistance during the COVID-19 pandemic by conducting PCR analysis of nasopharyngeal swabs for SARS-CoV-2. As a result, samples from patients across Ecuador were received. Those who provided informed consent to participate in subsequent studies and completed the relevant surveys were included in the study. A total of 10,175 individuals were included in this study. The cases were collected from different provinces of the Ecuadorian territory according to the samples arriving at the laboratory for biomedical sciences at ESPOL University. There were five main provinces from where the samples were obtained: Guayas, Manabí, Napo, Santa Elena, and Cotopaxi.

### Pharmacological aspects

Participants were asked about the consumption of drugs by a trained interviewer supervised by a health professional, Drugs were classified into main pharmacological groups: corticosteroids; antihistamines; mucolytics; multivitamins (including Vit C and Vit B; NSAIDs; acetaminophen). Other treatments recorded were chloride Dioxide and vaporisation. COVID-19-related symptoms drugs that were recorded were ivermectin, lopinavir or ritonavir, azithromycin, chloroquine, and hydroxychloroquine.

### PCR testing

Nasopharyngeal swab samples were collected and transported to the Laboratory for Biomedical Sciences at ESPOL University in Guayaquil according to the standard operational guidelines for response to coronavirus (COVID-19) of the Ministry of Public Health from Ecuador (MSP, 2020).

The nucleic acid extraction from samples magnetic-bead technology was used and RT-qPCR diagnostics were performed using the enzyme TaqPath™ 1-Step Multiplex Master Mix (No ROX) (ThermoFisher) and QuantStrudio 1 real-time PCR thermocycler.

Specific primers and probes targeting highly conserved genes such as N and ORF1b_Nsp14 were utilized for diagnosing the SARS-CoV-2 virus (Supplementary Table S1) (Centers for Disease Control and Prevention, 2020; Chu et al., 2020). In addition, reagents and sequences recommended by the Centers for Disease Control and Prevention (CDC) during the COVID-19 pandemic, as outlined in the document titled “CDC 2019-Novel Coronavirus (2019-nCoV) Real-Time RT-PCR Diagnostic Panel” published in March 2020, were employed for this study (Centers for Disease Control and Prevention, 2020).

For the RT-qPCR reaction, 5 μL of RNA served as the template in a final volume of 15 μL. The reaction followed the following program: reverse transcriptase (RT) was performed at 50°C for 20 min, followed by polymerase activation at 95°C for 2 min, and then subjected to 45 cycles at 95°C for 15 s and 60°C for 30 s.

The diagnostic test implemented in the laboratory involved real-time RT-PCR to qualitatively detect the nucleic acid of the SARS-CoV-2 virus from nasopharyngeal swabs. Quality controls comprised synthetic RNAs as positive controls and nuclease-free water as negative control. These controls underwent analysis in the same process as the patient samples. Additionally, the RnaseP gene was utilized as an internal positive control to monitor the extraction and RT-qPCR procedures.

A clinical sample was classified as positive for SARS-CoV-2 if the Ct value of both viral genes was ≤35. A negative result was assigned when the clinical sample showed no RNA amplification, and all controls exhibited the expected performance. In cases where only one of the two viral targets exhibited amplification or Ct values were ≥35.5, the results were deemed inconclusive, and testing was repeated. This included repeating nucleic acid extraction and performing repeated real-time RT-PCR. The effectiveness of the implemented real-time RT-PCR testing of the samples was compared with the nucleic acid from clinical respiratory samples obtained from a hospital centre. These samples had been previously analyzed using the commercial diagnostic kit TaqPath™ COVID-19 CE-IVD. Of 57 clinical respiratory samples that tested positive with the TaqPath™ COVID-19 CE-IVD kit, 56 were confirmed by the laboratory’s diagnostic panel, resulting in a positive agreement rate of approximately 98%.

### Statistical analysis

The chi-square test of independence compared clinical and demographic data and PCR test results. For 2 × 2 contingency tables, Fisher’s exact test is used instead. Similarly, Fisher’s exact test compares reported symptomatology and PCR test results. For the drug consumption dynamic analysis, odds ratios of drug consumption with a positive PCR result to a negative one was calculated for each pharmacological group. Significance was set as *p*-value <.05. All statistical analyses were performed using statistical software (R version 4.1.1).

## Results

### Study population and baseline characteristics

**FIGURE 2:**
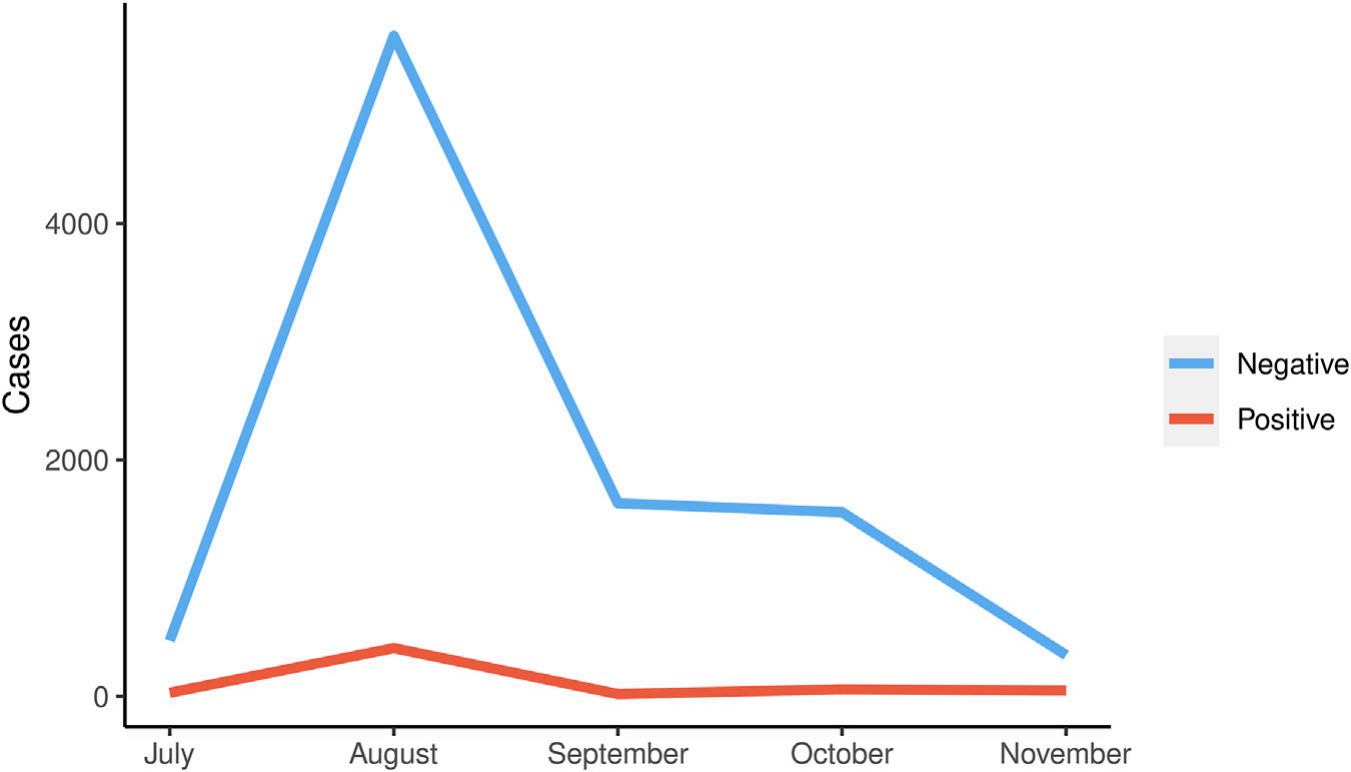
Distribution of the number of positive and negative cases during the first wave in Ecuador.

Our lab tested patients for SARS-CoV-2 during the first wave of the infection in Ecuador from July–November 2020. There were fewer positive cases tested at the laboratory during the first wave. There was a peak of positive cases in August, with 409 positive PCR (6.81% of the PCR test performed in August). We observed a decrease in positive cases in the coming months, Figure 2.

From all 10,175 samples, 570 were positive for SARS-CoV-2, and 9,605 were negative during the mentioned period in Ecuador, considering the provinces that sent the samples to the laboratory. This number corresponds to 5% of positive cases with no statistical association between the RT-PCR result and sex, age, or comorbidities.

Most of the samples tested were from adults between 18 and 65 years old, although significance was not observed concerning age. Comparing the differences between provinces, we observed significance (Table 1). The highest positive cases were from Cotopaxi (25.7%) and Napo (18.8%). The clinical antecedents, patients with autoimmune diseases (7.8%), cardiovascular (4.4%), and diabetes (6.3%) were more likely to be positive for SARS-CoV-2 even though there was no signficance.

**TABLE 1:** Demographic data from the population included in this study, according to positive and negative results from RT-PCR: quantity and proportion (%).

	PCR-positive N (%)	PCR-negative N (%)	*p*-*value*
*Gender*			
Female	299 (6.0)	4,657 (94.0)	0.074
Male	271 (5.2)	4,948 (94.8)	
*Age (years)*			
Infants: 0–5	3 (13.6)	19 (86.4)	
Children: 5–18	28 (5.7)	465 (94.3)	0.909^*^
Adults: 18–65	505 (5.6)	8,556 (94.4)	
Seniors >65	34 (5.7)	565 (94.3)	
*Provinces*			
Cotopaxi	44 (25.7)	127 (74.3)	
Guayas	150 (3.0)	4,844 (97.0)	
Manabí	214 (7.8)	2,537 (92.2)	
Napo	99 (18.8)	427 (81.2)	0.000
Santa Elena	59 (4.1)	1,379 (95.9)	
Other provinces	4 (1.4)	291 (98.6)	
*Clinical antecedents*			
Diabetes or/and Cardiovascular	22 (4.8)	436 (95.2)	0.508
None	540 (5.7)	8,986 (94.3)	
Other antecedents	8 (4.2)	183 (95.8)	

* For an appropriate approximation of the chi-square test, observations of infants and children were united in the same category.

From all 10,175 individuals, 36.5% reported symptoms with statistically higher symptomatic individuals among the PCR positive cases (53.2%) than the negative cases (35.5%) with a *p*-value <0.001. Analyzing each symptom separately, all reported symptoms but sneezing, nasal congestion, and skin rash show statistical dependency with RT-PCR results. Positive cases have reported these symptoms in a higher proportion than negative cases (Table 2). In absolute frequency, the most common symptoms within positive cases were cough (31.9%), fever (30.4%), and headache (25.8%).

**TABLE 2:** Reported symptomatology according to positive and negative results from RT-PCR: quantity and proportion (%).

	PCR-positive N (%)	PCR-negative N (%)	*p*-*value*
*Fever*			
Yes	173 (30.4)	1,516 (15.8)	0.000
No	397 (69.6)	8,089 (84.2)	
*Cough*			
Yes	182 (31.9)	1832 (19.1)	0.000
No	388 (68.1)	7,773 (80.9)	
*Shortness of breath*			
Yes	52 (9.1)	417 (4.3)	0.000
No	518 (90.9)	9,188 (95.7)	
*Tiredness*			
Yes	58 (10.2)	602 (6.3)	0.001
No	512 (89.9)	9,003 (93.7)	
*Sneezing*			
Yes	45 (7.9)	690 (7.2)	0.506
No	525 (92.1)	8,915 (92.8)	
*Nasal congestion*			
Yes	41 (7.2)	604 (6.3)	0.376
No	529 (92.8)	9,001 (93.7)	
*Sore throat*			
Yes	129 (22.6)	1,320 (13.7)	0.000
No	441 (77.4)	8,285 (86.3)	
*Headeache*			
Yes	147 (25.8)	1,411 (14.7)	0.000
No	423 (74.2)	8,194 (85.3)	
*Body pain*			
Yes	96 (16.8)	838 (8.7)	0.000
No	474 (83.2)	8,767 (91.3)	
*Diarrhea/abdominal discomfort*			
Yes	57 (10.0)	519 (5.4)	0.000
No	513 (90.0)	9,086 (94.6)	
*Loss of smell*			
Yes	50 (8.8)	306 (3.2)	0.000
No	520 (91.2)	9,299 (96.8)	
*Loss of taste*			
Yes	39 (6.8)	272 (2.8)	0.000
No	531 (93.2)	9,333 (97.2)	
*Skin rash*			
Yes	12 (2.1)	266 (2.8)	0.427
No	558 (97.9)	9,339 (97.2)	

### Prevalence of COVID-19 cases in Ecuador

**FIGURE 3:**
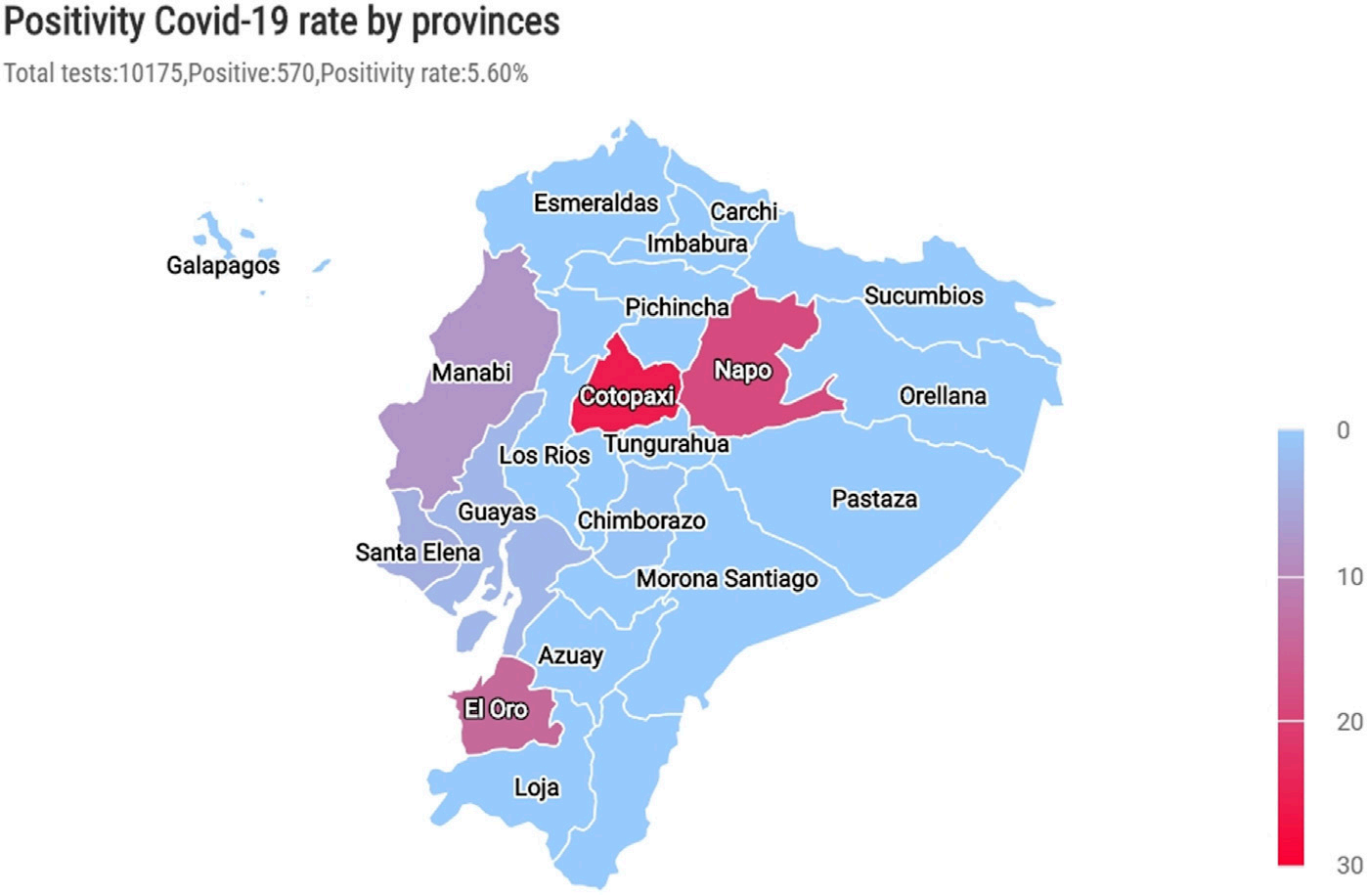
The prevalence of positive cases by provinces during the first wave. The prevalence of positive cases within each province is according to the total number of tests in the same area.

The analysis of COVID-19 prevalence based on the provinces assisted in comprehending the proportion of positive cases in different areas (Figure 3). Cotopaxi and Napo had the highest rates of positive cases (25.7% and 18.8%, respectively, Table 1, Figure 3), whereas Manabí, Santa Elena, and Guayas had fewer than 10% of positive cases (7.8%, 4.1%, and 3.0%, respectively, Table 1, Figure 3).

### Drug consumption dynamic

**FIGURE 4:**
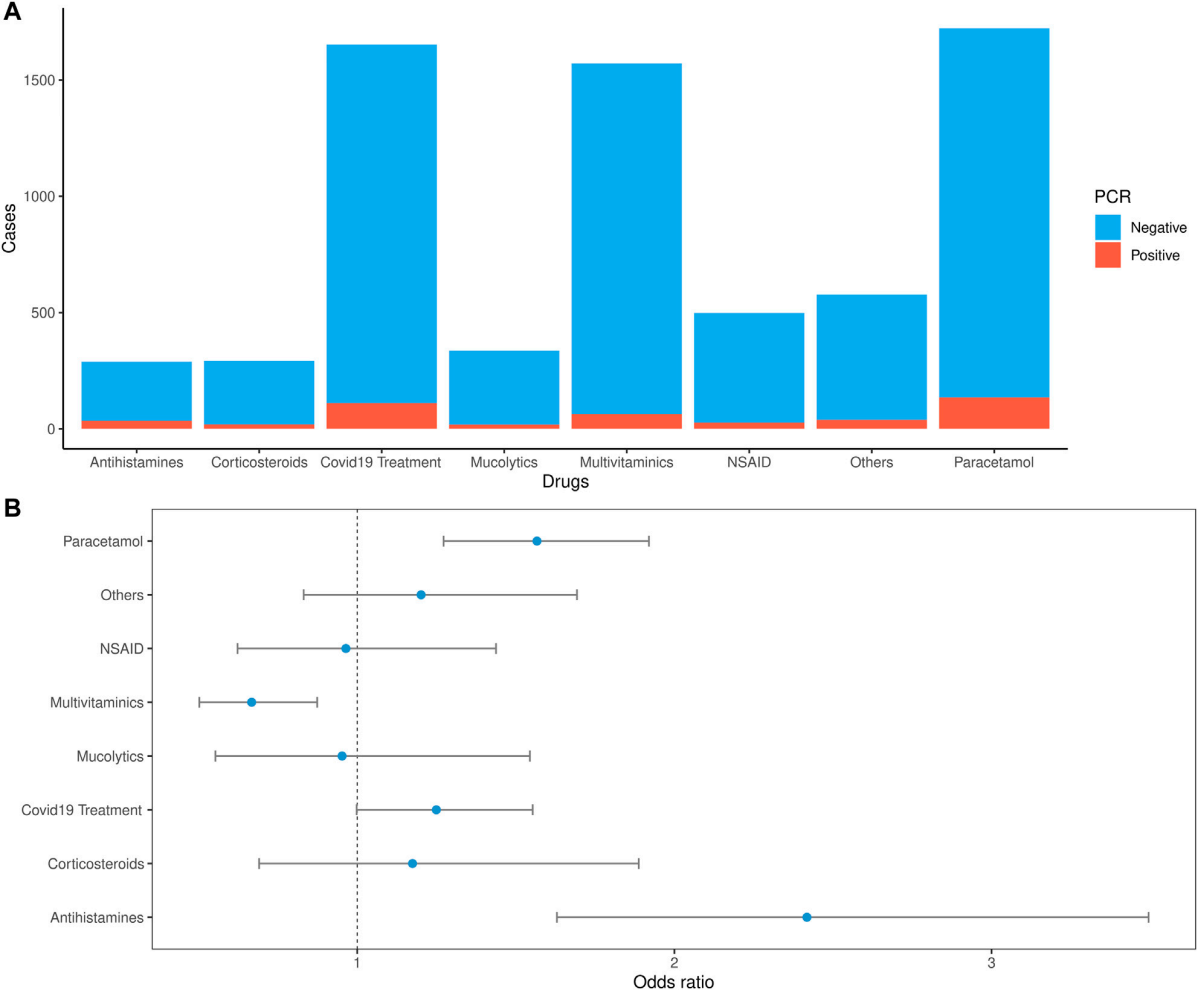
Drug consumption dynamics of the COVID-19 PCR test. **(A)**. Percentages out of the total cases vs. drug consumption by the PCR test results **(B)**. Odds ratio: the odds of each drug consumption in PCR positive group *versus* the odds of each drug consumption in PCR negative group with 95% confidence intervals.

**FIGURE 5:**
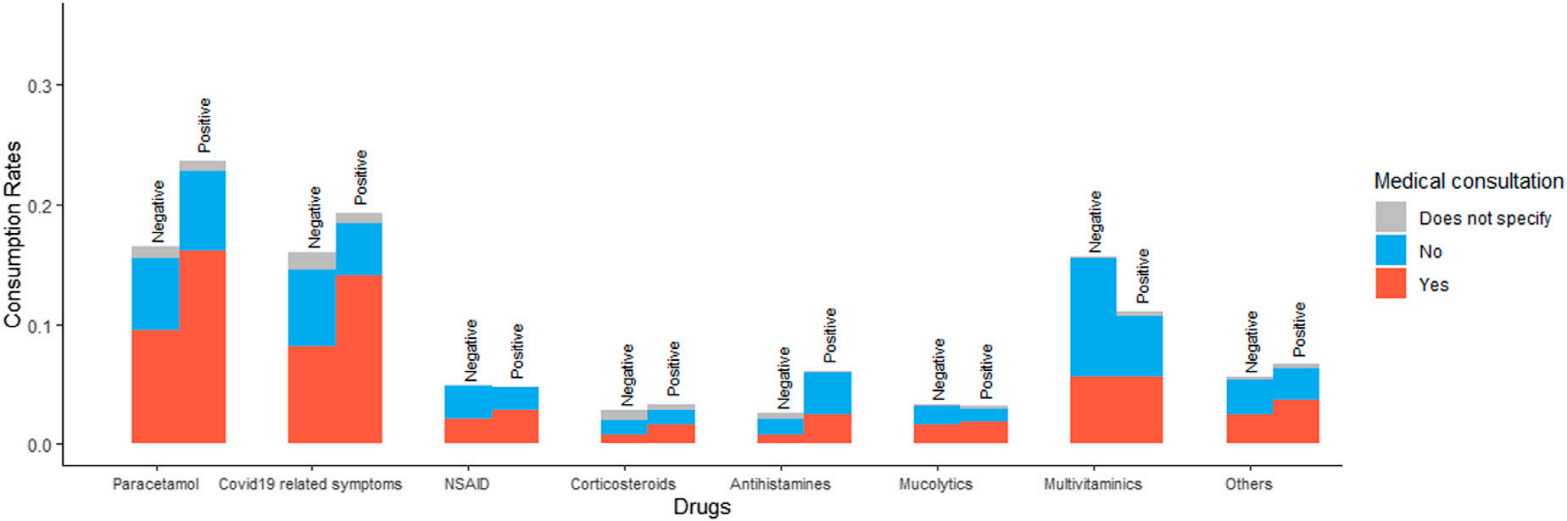
Percentage of medical consultation by each drug consumption relative to the total number of PCR positive or negative individuals.

We compared the drug consumption dynamic, including the number of cases, drugs, and PCR results of the participants. Overall, out of all participants, 31.3% reported the consumption of at least one drug with no statistical difference between the PCR positive (32.1%) and negative (31.2%) cases (*p*-value: 0.676). Analyzing each treatment category, the most consumed treatment was acetaminophen (16.9%) and COVID-19-related symptoms drugs (16.2%), Figure 4A. The odds of consumption of acetaminophen and antihistamines in PCR positive group were statistically higher than the odds of consumption of those drugs in PCR negative group (95% confidence intervals for odds ratio only include values larger than 1, Figure 4B). In contrast, multivitamins had higher odds of consumption among patients with negative PCR results than positive patients. There was no difference in the odds of consumption between the two groups for Corticosteroids, Mucolytics, and NSAIDs. Additionally, 53.4% of participants survey who reported consumption of at least one drug was self-medicated. The results show that self-medication incidence does not differ between PCR-results, except for multivitamins’ which was higher on the PCR negative group (Figure 5).

## Discussion

This retrospective study provides detailed information about SARS-CoV-2 and the drug consumption dynamics during the first wave of the COVID-19 pandemic in Ecuador. ESPOL reported 409 positive cases, with a prevalence of 5.7% of positive cases for SAR-CoV-2 from July to November 2020, at a national level. Our study found that Cotopaxi and Napo had 26% and 19% positive case rates, respectively, although official data indicate that these provinces only presented less than 2% of cases in Ecuador (Observatorio Social del Ecuador, 2022). Another study that referred exclusively to the Manabí province showed a prevalence of 23.7% of cases in the first wave. Similarly, we have found a prevalence of 20% of COVID-19 in this province, even though other provinces have shown a major impact (Ortiz-Prado et al., 2021). In a study conducted during the initial wave of the COVID-19 pandemic, Morales-Jadan et al. aimed to determine the infection rate of SARS-CoV-2 in rural communities belonging to four provinces in the Andean region of Ecuador. They conducted RT-qPCR testing on 1,021 individuals and obtained an overall infection rate of 26.2%, with 268 positive cases. These findings are similar to our results, which showed an overall infection rate of 22%. Morales-Jadan et al. also reported that Tungurahua province had the highest infection rate at 65.3%, followed by Chimborazo at 16.77%, the mountainous areas of Napo at 38.2%, and Bolívar at 5.1%. Notably, several communities had an infection rate of over 50% (Morales-Jadán et al., 2023). The viral load in communities such as Penipe, Pelileo, El Chaco, and Riobamba exceeded 10^8 copies/mL, representing 7.46% of the infected population. This rate is similar to our findings of a 5.7% prevalence of infection. In Esmeraldas, 1,259 individuals across the municipalities of Esmeraldas, Atacames, Muisne, Quinindé, and Río Verde were subjected to RT-qPCR and anti-SARS-CoV-2 IgG serological tests. The overall infection rate was 7.7% and 11.68% based on RT-qPCR and IgG seroprevalence, respectively. The highest infection rates by the community were in Quinindé (12.17%) based on RT-qPCR samples and Atacames with a rate of 24.47% based on IgG seroprevalence (Vallejo-Janeta et al., 2022).

Comorbidities are a well-known risk factor for second for more severe symptoms during COVID-19 (Cuschieri and Grech, 2020; Ma and Holt, 2020; Kaur et al., 2021). Our study did not determine the prevalence of diabetes or cardiovascular diseases in infected patients. Diabetes is a comorbidity that affected more than 430 million worldwide in 2019 and has been unfavourably associated with the natural history of the virus and with an overall increased risk of infection resulting from multiple disturbances of innate immunity (Cuschieri and Grech, 2020; Ma and Holt, 2020). Cardiovascular comorbidities predispose to suffer up to three times more severe forms of COVID-19 (Salazar et al., 2020; Greenberg et al., 2021). There is an increased risk of acute infarction of the myocardium, myocarditis, heart failure, shock, arrhythmias, and sudden death We estimated the relationship between them (Alyammahi et al., 2021; Greenberg et al., 2021). We showed a higher correlation between Diabetes, Cardiovascular diseases, and autoimmune diseases with positive RT-PCR results.

Additionally, we determined whether there were any risks considering the age and sex. Guan and collaborators and Voinsky and collaborators indicated a correlation between age and PCR-positivity with age over 30 years presenting a higher risk of positivity (Lai et al., 2020; Voinsky et al., 2020). There was no correlation between the age and sex between positive/relation between positive/negative PCR, different from as reported before because the significant population in our study was adults. There were not many infants, adolescents, and seniors.

Our group has previously reported that 55% of the Ecuadorian population was at a high risk of self-medication during the first wave of confinement (Orellana Manzano et al., 2021). Our results corroborate this, showing that 50% of Ecuadorians surveyed self-medicated without medical consultation. This study shows that paracetamol was the most consumed drug during the first wave. Moreover, it is the most used medication worldwide. It has been abused in advance during COVID-19 (Sestili and Fimognari, 2020; Orellana Manzano et al., 2021; Pandolfi et al., 2021; Domingo-Echaburu et al., 2022). In a study conducted in Santo Domingo, students from the Pontificia Universidad Católica del Ecuador diagnosed with COVID-19 received acetaminophen and azithromycin using acetaminophen and azithromycin medical prescription, mainly after perceiving symptoms such as loss of smell, fever, and fatigue. Considering the COVID-19-related symptoms drugs options during the first wave, such as ivermectin, lopinavir or ritonavir, azithromycin, chloroquine, and hydroxychloroquine, there were no correlations between COVID-19 diagnosis and symptoms or medical appointment, although these drugs were extensively used. This result indicates a public health concern because of the impact of this type of self-medication on health and the economy (Vargas Patiño, 2021).

Hydroxychloroquine, ivermectin, and azithromycin as self-medication for COVID-19 remain controversial and potentially harmful, despite their known antimalarial, anti-inflammatory, anti-infectious, and immunomodulatory properties (Baracaldo-Santamaría et al., 2022). Clinical trials have shown that hydroxychloroquine provides no clinical benefit for prophylaxis in non-hospitalized and hospitalized patients with COVID-19 (Ricardo Martins-Filho et al., 2021). Safety concerns include gastrointestinal adverse reactions, cardiotoxicity, and ocular toxicity. Similarly (Baracaldo-Santamaría et al., 2022), due to limited and non-standardized studies, the efficacy and safety of ivermectin for COVID-19 treatment still need to be determined. Adverse reactions to using ivermectin include abdominal pain, diarrhea, and taste alterations (Mohan et al., 2021). Azithromycin therapy in COVID-19 treatment can also lead to adverse effects, such as gastrointestinal distress, hepatotoxicity, and hypersensitivity reactions (Oshikoya et al., 2019; Zhao et al., 2022). The main concern is the risk of QTc interval prolongation, which can lead to adverse cardiovascular effects (Ramireddy et al., 2020; Zhao et al., 2022). Therefore, these drugs should only be used under close medical supervision, and self-medication should be avoided. It is important to note that the FDA and other institutions advise against using these drugs for COVID-19 until conclusive studies support their use (FDA, 2020; BJC HealthCare, 2023).

Self-medication (SM) has become a prevalent global health trend, particularly during the COVID-19 pandemic although it can have serious implications. It may mask symptoms and delay accurate diagnosis, besides contributing to developing resistance to antibiotics, antivirals, and ivermectin, compromising the effectiveness of these treatments against their respective diseases. Other concerns of SM include are adverse reactions, drug interactions, incorrect administration and dosage, inappropriate choice of therapy, masking of severe diseases, and the risk of dependence and abuse. In Ecuador, few research groups have statistically demonstrated the increase of SM during the COVID-19 outbreak. A study conducted in Quito aimed to identify medication consumption patterns and their prevalence during the first year of confinement. A total of 401 online questionnaires were completed, with 60.9% of respondents indicating that their objective for consuming medication was to treat an infection. However, 118 individuals, representing 48.8% of the respondents, practised self-medication for either prevention or treatment. These individuals obtained information from external and non-medical sources such as the Internet (79.5%), social media (78.3%), television/radio (81.3%), and advice from family members (84.4%). The most used medications were paracetamol (87.3%), ibuprofen (47.5%), and azithromycin (91.4% as treatment and 8.6% as prevention) (Arias et al., 2022). During the initial wave of the COVID-19 pandemic, azithromycin was suggested as a treatment for patients with severe virus cases who did not exhibit bacterial superinfection. Unfortunately, this recommendation led to its intentional consumption, elevating the risk of adverse effects. Subsequently, in September 2020, the Consensus Recommendations for Ambulatory Management and Home Treatment of COVID-19 patients withdrew the recommendation due to the associated concerns (Yang et al., 2020; Ministerio de Salud Pública, 2021). These reports are like our results, in which there is a high risk of SM in patients with positive COVID-19 PCR. The motivations of these SM practices must be studied to examine the impact of SM on public health. Further research is warranted in Ecuador to determine the influence of SM on COVID-19 prevention and treatment.

## Data Availability

The raw data supporting the conclusion of this article will be made available by the authors, without undue reservation.

## References

[B1] Agencia Nacional de Regulación C. V. S. (2020). Resolución ARCSA-DE-002-2020-LDCL la dirección ejecutiva de la agencia nacional de regulación, control Y vigilancia sanitaria-arcsa. Guayaquil, Ecuador: ARCSA.

[B2] Alyammahi S. K. Abdin S. M. Alhamad D. W. Elgendy S. M. Altell A. T. Omar H. A. (2021). The dynamic association between COVID-19 and chronic disorders: An updated insight into prevalence, mechanisms and therapeutic modalities. Infect. Genet. Evol. 87, 104647. 10.1016/J.MEEGID.2020.104647 33264669 PMC7700729

[B3] Arias F. Izquierdo-Condoy J. S. Naranjo-Lara P. Alarcón V. Bonilla P. Erazo E. (2022). A cross-sectional analysis of self-medication patterns during the COVID-19 pandemic in Ecuador. Med. (B Aires) 58, 1678. 10.3390/medicina58111678 PMC9698278 36422217

[B4] Baracaldo-Santamaría D. Pabón-Londoño S. Rojas-Rodriguez L. C. (2022). Drug safety of frequently used drugs and substances for self-medication in COVID-19. Ther. Adv. Drug Saf. 13, 20420986221094141. 10.1177/20420986221094141 35493401 PMC9039440

[B5] Bennadi D. (2013). Self-medication: A current challenge. J. Basic Clin. Pharm. 5, 19–23. 10.4103/0976-0105.128253 24808684 PMC4012703

[B6] BJC HealthCare (2023). Pharmacologic prevention and management of non-hospitalized adults with COVID-19* BJC COVID-19 outpatient treatment options see table 2 for more information and BJC criteria for use pre-exposure prophylaxis.

[B7] Centers for Disease Control and Prevention (2020). Specific primers and probes for detection 2019 novel coronavirus. China.

[B8] Chu D. K. W. Pan Y. Cheng S. M. S. Hui K. P. Y. Krishnan P. Liu Y. (2020). Molecular diagnosis of a novel coronavirus (2019-nCoV) causing an outbreak of pneumonia. Clin. Chem. 66, 549–555. 10.1093/clinchem/hvaa029 32031583 PMC7108203

[B9] Cuschieri S. Grech S. (2020). COVID-19 and diabetes: The why, the what and the how. J. Diabetes Complicat. 34, 107637. 10.1016/J.JDIACOMP.2020.107637 PMC7242955 32456846

[B10] Domingo-Echaburu S. Irazola M. Prieto A. Rocano B. Lopez de Torre-Querejazu A. Quintana A. (2022). Drugs used during the COVID-19 first wave in Vitoria-Gasteiz (Spain) and their presence in the environment. Sci. Total Environ. 820, 153122. 10.1016/j.scitotenv.2022.153122 35063509 PMC8767721

[B11] Dyer O. (2020). Covid-19: Remdesivir has little or no impact on survival, WHO trial shows. BMJ 371, m4057. 10.1136/BMJ.M4057 33077424

[B12] FDA (2020). FDA cautions against use of hydroxychloroquine or chloroquine for COVID-19 outside of the hospital setting or a clinical trial due to risk of heart rhythm problems | FDA. FDA. Available at: https://www.fda.gov/drugs/drug-safety-and-availability/fda-cautions-against-use-hydroxychloroquine-or-chloroquine-covid-19-outside-hospital-setting-or (Accessed May 8, 2023).

[B13] Greenberg A. Pemmasani G. Yandrapalli S. Frishman W. H. (2021). Cardiovascular and cerebrovascular complications with COVID-19. Cardiol. Rev. 29, 143–149. 10.1097/CRD.0000000000000385 33758123 PMC8021013

[B14] Hughes C. M. McElnay J. C. Fleming G. F. (2001). Benefits and risks of self medication. Drug Saf. 24, 1027–1037. 10.2165/00002018-200124140-00002 11735659

[B15] Kaur H. Thakur J. S. Paika R. Advani S. M. (2021). Impact of underlying comorbidities on mortality in SARS-COV-2 infected cancer patients: A systematic review and meta-analysis. Asian Pac. J. Cancer Prev. 22, 1333–1349. 10.31557/APJCP.2021.22.5.1333 34048161 PMC8408376

[B16] Lai C.-C. Liu Y. H. Wang C.-Y. Wang Y.-H. Hsueh S.-C. Yen M.-Y. (2020). Asymptomatic carrier state, acute respiratory disease, and pneumonia due to severe acute respiratory syndrome coronavirus 2 (SARSCoV-2): Facts and myths. J. Microbiol. Immunol. Infect. 2, 404–412. 10.1016/j.jmii.2020.02.012 PMC7128959 32173241

[B18] Ma R. C. W. Holt R. I. G. (2020). COVID‐19 and diabetes. Diabet. Med. 37, 723–725. 10.1111/DME.14300 32242990 PMC7228343

[B19] Ministerio de Salud Pública (2021). Consenso Multidisciplinario informado en la evidencia sobre el tratamiento de Covid-19 – Ministerio de Salud Pública. MSP. Available at: https://www.salud.gob.ec/consenso-multidisciplinario-informado-en-la-evidencia-sobre-el-tratamiento-de-covid-19/ (Accessed May 22, 2023).

[B21] Mohan A. Tiwari P. Suri T. M. Mittal S. Patel A. Jain A. (2021). Single-dose oral ivermectin in mild and moderate COVID-19 (RIVET-COV): A single-centre randomized, placebo-controlled trial. J. Infect. Chemother. 27, 1743–1749. 10.1016/J.JIAC.2021.08.021 34483029 PMC8384587

[B22] Molento M. B. (2020). COVID-19 and the rush for self-medication and self-dosing with ivermectin: A word of caution. One Health 10, 100148. 10.1016/J.ONEHLT.2020.100148 32632377 PMC7313521

[B23] Montaño D. (2020). Covid-19 en Ecuador: Indígenas enfrentan el riesgo de la automedicación. *Mongabay* . Available at: https://es.mongabay.com/2020/07/covid-19-ecuador-indigenas-automedicacion/ (Accessed September 20, 2022).1.

[B24] Morales-Jadán D. Vallejo-Janeta A. P. Bastidas V. Paredes-Espinosa M. B. Freire-Paspuel B. Rivera-Olivero I. (2023). High SARS-CoV-2 infection rates and viral loads in community-dwelling individuals from rural indigenous and mestizo communities from the Andes during the first wave of the COVID-19 pandemic in Ecuador. Front. Med. (Lausanne) 10, 1001679. 10.3389/FMED.2023.1001679 36844208 PMC9949717

[B25] MSP (2020). Lineamientos Operativos de respuesta frente a coronavirus COVID-19. Available at: https://www.salud.gob.ec/wp-content/uploads/2020/03/lineamiento-operativo-coronavirus-FINAL_02-2020.pdf .

[B26] Observatorio Social del Ecuador (2022). Provincias | coronavirus Ecuador. Observatorio Social del Ecuador. Available at: https://www.covid19ecuador.org/provincias (Accessed July 4, 2022).

[B27] Onchonga D. Omwoyo J. Nyamamba D. (2020). Assessing the prevalence of self-medication among healthcare workers before and during the 2019 SARS-CoV-2 (COVID-19) pandemic in Kenya. Saudi Pharm. J. 28, 1149–1154. 10.1016/J.JSPS.2020.08.003 32837218 PMC7426227

[B28] Orellana Manzano A. K. Orellana Manzano S. Dorado Sanchez L. Vizcaino M. J. Gomez‐Franco F. Chuquimarca‐Tandazo L. (2021). Self‐medication risk during SARS‐COV‐2 confinement pandemic. FASEB J. 35, 04814. 10.1096/fasebj.2021.35.S1.04814 PMC8239554 34318948

[B29] Ortiz-Prado E. Henriquez-Trujillo A. R. Rivera-Olivero I. A. Freire-Paspuel B. Vallejo-Janeta A. P. Lozada T. (2021). Massive SARS-CoV-2 RT-PCR testing on rural communities in manabi province (Ecuador) reveals severe COVID-19 outbreaks. Am. J. Trop. Med. Hyg. 104, 1493–1494. 10.4269/AJTMH.20-1208 33556041 PMC8045655

[B30] Oshikoya K. A. Wharton G. T. Avant D. Van Driest S. L. Fenn N. E. Lardieri A. (2019). Serious adverse events associated with off-label use of azithromycin or fentanyl in children in intensive care units: A retrospective chart review. Pediatr. Drugs 21, 47–58. 10.1007/s40272-018-0318-9 PMC6387682 30484072

[B31] Pandolfi S. Simonetti V. Ricevuti G. Chirumbolo S. (2021). Paracetamol in the home treatment of early COVID‐19 symptoms: A possible foe rather than a friend for elderly patients? J. Med. Virol. 93, 5704–5706. 10.1002/JMV.27158 34170556 PMC8426871

[B32] Quincho-Lopez A. Benites-Ibarra C. A. Hilario-Gomez M. M. Quijano-Escate R. Taype-Rondan A. (2021). Self-medication practices to prevent or manage COVID-19: A systematic review. PLoS One 16, e0259317. 10.1371/JOURNAL.PONE.0259317 34727126 PMC8562851

[B33] Quispe-Cañari J. F. Fidel-Rosales E. Manrique D. Mascaró-Zan J. Huamán-Castillón K. M. Chamorro–Espinoza S. E. (2021). Self-medication practices during the COVID-19 pandemic among the adult population in Peru: A cross-sectional survey. Saudi Pharm. J. 29, 1–11. 10.1016/J.JSPS.2020.12.001 33519270 PMC7832015

[B34] Ramireddy A. Chugh H. Reinier K. Ebinger J. Park E. Thompson M. (2020). Experience with hydroxychloroquine and azithromycin in the coronavirus disease 2019 pandemic: Implications for qt interval monitoring. J. Am. Heart Assoc. 9, e017144. 10.1161/JAHA.120.017144 32463348 PMC7429030

[B35] Ricardo Martins-Filho P. Campos Ferreira L. Heimfarth L. Antunes de Souza Araújo A. José Quintans-Júnior L. Ricardo P. (2021). Efficacy and safety of hydroxychloroquine as pre-and post-exposure prophylaxis and treatment of COVID-19: A systematic review and meta-analysis of blinded, placebo-controlled, randomized clinical trials. Lancet Regional Health - Am. 2, 100062. 10.1016/j.lana.2021.100062 PMC8403035 34485970

[B36] Salazar M. Barochiner J. Espeche W. Ennis I. (2020). [COVID-19 and its relationship with hypertension and cardiovascular disease]. Hipertens. Riesgo Vasc. 37, 176–180. 10.1016/J.HIPERT.2020.06.003 32591283 PMC7301092

[B37] Sestili P. Fimognari C. (2020). Paracetamol use in COVID-19: Friend or enemy?

[B38] Torijano Casalengua M. L. Calvo Pita C. Maderuelo-Fernández J. Á. (2021). A safe use of medications in Primary Care, in COVID-19 pandemic as well. Aten. Primaria 53, 102223. 10.1016/j.aprim.2021.102223 34961581 PMC8708816

[B39] Vallejo-Janeta A. P. Morales-Jadan D. Paredes-Espinosa M. B. Coronel B. Galvis H. Bone-Guano H. R. (2022). Sustained COVID-19 community transmission and potential super spreading events at neglected afro-ecuadorian communities assessed by massive RT-qPCR and serological testing of community dwelling population. Front. Med. (Lausanne) 9, 2326. 10.3389/fmed.2022.933260 PMC9433781 36059834

[B40] Vargas Patiño K. N. (2021). Factores asociados a la automedicación y consumo de medicamentos durante la pandemia del coronavirus (SARS-COV2) en países de América Latina. Available at: https://repositorio.continental.edu.pe/handle/20.500.12394/10475 (Accessed July 4, 2022).

[B41] Voinsky I. Baristaite G. Gurwitz D. (2020). Effects of age and sex on recovery from COVID-19: Analysis of 5769 Israeli patients. J. Infect. 81, e102–e103. 10.1016/j.jinf.2020.05.026 PMC7229949 32425274

[B42] World Health Organization (WHO) (2020). WHO in-house assays COVID-19 RT-qPCR 202.

[B43] Yang X. Yu Y. Xu J. Shu H. Xia J. Liu H. (2020). Clinical course and outcomes of critically ill patients with SARS-CoV-2 pneumonia in wuhan, China: A single-centered, retrospective, observational study. Lancet Respir. Med. 8, 475–481. 10.1016/S2213-2600(20)30079-5 32105632 PMC7102538

[B44] Zhao Y. Zhang J. Zheng K. Thai S. Simpson R. J. Kinlaw A. C. (2022). Serious cardiovascular adverse events associated with hydroxychloroquine/chloroquine alone or with azithromycin in patients with COVID-19: A pharmacovigilance analysis of the FDA adverse event reporting system (faers). Drugs Real World Outcomes 9, 231–241. 10.1007/s40801-022-00300-y 35386046 PMC8985751

