# Supplementary material for: A report on SARS-CoV-2 first wave in Ecuador: drug consumption dynamics
Source: Front Pharmacol. 2023 Jun 14;14:1197973. doi: 10.3389/fphar.2023.1197973 (PMC10300276; doi:10.3389/fphar.2023.1197973)
Supplement: Supplementary file 1 [file Table1.docx]

**Supplementary table 1:**  Primer and probe oligonucleotides used for RT-qPCR assays.

| **Virus** | **Target** | **Function** | **Primer/**  **probe** | **Sequence (5'-3')** |
| --- | --- | --- | --- | --- |
| SARS-CoV-2 | Nucleoprotein gene | Forward primer | 2019-nCoV_N1-F | GACCCCAAAATCAGCGAAAT |
|  |  | Reverse primer | 2019-nCoV_N1-R | TCTGGTTACTGCCAGTTGAATCTG |
|  |  | Probe | 2019-nCoV_N1-Probe | ROX-ACCCCGCATTACGTTTGGTGGACC-3’-BHQ2 |
|  | ORF1b-nsp14 gene | Forward primer | HKU-nsp14F | TGGGGYTTTACRGGTAACCT |
|  |  | Reverse primer | HKU-nsp14R | AACRCGCTTAACAAAGCACTC |
|  |  | Probe | HKU-nsp14-Probe | FAM-TAGTTGTGATGCWATCATGACTAG-3’-BHQ1 |
| Endogenous human gene control | RnaseP gene | Forward primer | RnaseP-F | AGATTTGGACCTGCGAGCG |
|  |  | Reverse primer | RnaseP-R | GAGCGGCTGTCTCCACAAGT |
|  |  | Probe | RnaseP-Probe | HEX-TTCTGACCTGAAGGCTCTGCGCG-3’-BHQ1 |

*Molecular Probes: ROX 6-Carboxy-X-Rhodamine, FAM 6-carboxyfluorescein, HEX Hexachlorofluorescein; Black Hole Quencher dyes: BHQ1 Black Hole Quencher 1 used with molecular probes FAM, and HEX and BHQ2 Black Hole Quencher 2 used with molecular probe ROX*
